# Improved ocular gene transfer with a Neddylation-site modified AAV-RPE65 vector in rd12 mice

**DOI:** 10.1038/s41433-020-0838-8

**Published:** 2020-03-09

**Authors:** Shubham Maurya, Bertin Mary, Giridhara R. Jayandharan

**Affiliations:** Department of Biological Sciences and Bioengineering, Indian Institute of Technology, Kanpur, India

**Keywords:** Retinal diseases, Biologics

Leber congenital amaurosis type 2 (LCA2) is an inherited disease that affects the integrity of the retina and results in severe visual impairment early in life [1]. This disease is caused due to mutations in the retinal pigmental epithelium (*RPE*) 65 kDa protein encoding gene, that generates an isomerohydrolase enzyme in the visual cycle [2]. Gene therapy is an attractive option for this condition, due to the relatively immune privileged nature of the eye. Adeno-associated virus serotype 2 (AAV2) vectors have been utilized to deliver *RPE65* gene into LCA2 patients [3]. The long-term follow-up data [4] demonstrated a peak functional rescue, ~1 year after gene therapy, but subsequently a decline in RPE65 expression and immune response was noted [5]. Thus, AAV2 vectors that can augment visual function at significantly lower doses are needed. In our recent study [6], we have identified and demonstrated the role of ubiquitin-like modifiers such as Neddylation in AAV2 vectors and abolition of these sites, augmented coagulation factor IX expression in hemophilia B mice.

**Fig. 1:**
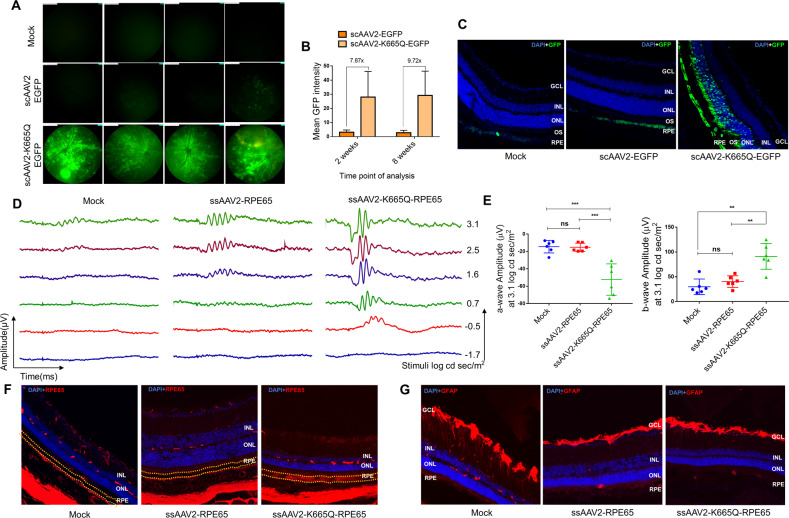
Ocular gene transfer in C57BL6/J and rd12 mice with a Neddylation-site modified K665Q vector. Eyes of C57BL6/J mice (*n* = 8) were mock injected or injected with scAAV2-EGFP and scAAV2-K665Q-EGFP vectors at a dose of 3 × 10^8^ vgs by either intravitreal or subretinal route. **a** Fundus imaging of the murine eyes was performed 2 and 8 weeks after intravitreal administration in a Micron IV imaging system (Phoenix research labs, CA, USA). Representative images from 8-week imaging data are shown. Intensity was set at maximum and gain was set at 15 db, frame rate was set at 6 fps, for imaging of all the groups. **b** A quantification of the data obtained was performed by using Concentric Circle Plugin in the ImageJ software. **c** Murine eyes administered *via* subretinal administration with AAV vectors were enucleated after 4 weeks and 8 µM cryosections were obtained. Sections of mock injected, scAAV2-EGFP, and scAAV2-K665Q-EGFP groups were counterstained for nuclei, with 4′,6-diamidino-2-phenylindole (DAPI). Fluorescence images were acquired by A1R HD25 Nikon Confocal (Ti-2 eclipse body) microscope (Tokyo, Japan) with a 405 nm and 488 nm laser equipped with Galvano scanners. Representative images are shown. **d** Eyes of rd12 mice (*n* = 6) were mock- injected or injected with ssAAV2-RPE65 and ssAAV2-K665Q-RPE65 vectors at a dose of 7 × 10^8^ vgs, *via* subretinal route. Scotopic electroretinography was performed after 16 weeks by a Ganzfeld ERG system (Phoenix research labs, CA, USA). Representative ‘A-wave’ and ‘B-wave’ forms are shown (**d**) along with the quantitative data (**e**). Data shown are mean ± SD. ANOVA based Dunett’s test was used for statistical comparison between the groups. **p < 0.01, ***p < 0.001. **f**, **g** For immunostaining, 8 µM cryosections of mock injected, ssAAV2-RPE65 and ssAAV2-K665Q-RPE65 groups were stained with anti-RPE65 (1:100, Abcam, Cambridge, UK) and counterstained with goat anti-mouse Alexa Fluor™ 555 (1:300, Abcam) antibody or with anti-GFAP (1:100, Cell signaling technologies, Danvers, USA) and counterstained with goat anti-rabbit cy3 antibody (1:300, Jackson ImmunoResearch, West Grove, USA). Images were acquired in Leica DMi8 confocal microscope (Wetzlar, Germany) with a 405 nm and 532 nm laser equipped with Galvano scanners. A representative set of DAPI overlay images with RPE65 (**f**)/GFAP (**g**) staining are shown. GCL ganglion cell layer, INL inner nuclear layer, ONL outer nuclear layer; OS outer segment; RPE retinal pigment epithelium (marked with dotted line in (**f**)).

The present study was designed to evaluate if these Neddylation-site modified AAV2 vectors are effective, during ocular gene therapy. To assess this, we packaged wild-type (WT) AAV2 vector (ssAAV2-RPE65; scAAV2-CB-EGFP) and a Neddylation-mutant vector containing a human *RPE65* gene or the enhanced green fluorescent protein (*EGFP*) gene (ssAAV2-K665Q-RPE65; scAAV2-K665Q-CB-EGFP), as described earlier [6]. Vector titers were measured by a quantitative PCR and are expressed as vector genomes (vgs)/ml. All the animal experiments were approved by the IIT-Kanpur animal ethics committee. The AAV vectors thus generated were assessed by in vivo ocular gene transfer by different routes of delivery (intravitreal and subretinal) and strains of mice [C57BL6/J and rd12, Jackson Laboratory (Bar Harbor, USA)]. In the first set of investigations, eyes (*n* = 8 per group) of C57BL6/J mice were either mock injected or injected with AAV2-WT-EGFP and AAV2-K665Q-EGFP vectors by the intravitreal or subretinal route at a dose of 3 × 10^8^ vgs/eye. Fluorescence imaging of the eyes was performed, 2 and 8 weeks after ocular gene transfer in a Micron IV imaging system (Phoenix Research Lab, Pleasanton, USA). Our data shown in Fig. 1a, demonstrate that the K665Q mutant had a significantly higher EGFP expression (7.87–9.72-fold, *p* < 0.05, Fig. 1b) when compared with eyes that were administered with AAV2-WT vectors, intravitreally.

We then examined the transduction potential of the AAV2-K665Q-EGFP vector by subretinal administration. Four weeks later, retinal sections (8 µm) of the murine eyes was prepared, stained with DAPI (Sigma-Aldrich, St. Louis, USA) and mounted with FluorSave™ (Sigma-Aldrich). Images were acquired in a confocal microscope (A1R HD25 Nikon, Tokyo, Japan). Our analysis of K665Q-EGFP vector administered eyes, revealed a markedly enhanced GFP expression within the RPE layer and the outer segment of the retina in comparison to AAV2-WT vector (Fig. 1c).

To further evaluate the therapeutic efficiency of the mutant AAV2 vectors in a murine model of retinal degeneration, we administered either PBS (mock group), AAV2-WT, and AAV2-K665Q vectors expressing human RPE65, in rd12 mice. Approximately, 1–2 µl of AAV vectors at dose of 7 × 10^8^ vgs was administered *via* subretinal route into the murine eyes (*n* = 6 eyes per group). The phenotypic response was measured by scotopic electroretinography (ERG), 16 weeks after vector administration (Ganzfeld ERG, Phoenix Research lab). The representative ERG waveforms from the treated mice are shown in Fig. 1d. We noted a significant visual correction in eyes that received AAV2-K665Q-RPE65 vectors, with a 2.43-fold (*p* < 0.001) increase in ‘A-wave’ amplitude and a 1.25-fold (*p* < 0.01) increase in ‘B-wave’ amplitude in comparison to the AAV2-WT injected animals (Fig. 1d, e), at the very low doses of AAV vectors administered in this study (7 × 10^8^ vgs/eye). Also, immunostaining of vector injected eye sections with an anti-RPE65 antibody (Abcam, Cambridge, UK) revealed enhanced RPE65 expression in AAV2-K665Q administered animals (Fig. 1f). Further, the expression of glial fibrillary acidic protein (GFAP) was similar between the treatment groups suggesting the lack of inflammation in treated eyes (Fig. 1g).

Our preclinical data presented here highlights the translational potential of Neddylation-site modified vectors for retinal gene therapy. However, further long-term follow-up data and a comprehensive evaluation of ocular immune response in vivo are required prior to its clincial application.
